# Crystal strucutre of *rac*-methyl (11aR*,12S*,13R*,15aS*,15bS*)-11-oxo-11,11a,12,13-tetra­hydro-9*H*,15b*H*-13,15a-ep­oxy­isoindolo[1,2-*c*]pyrrolo[1,2-*a*][1,4]benzodiazepine-12-carboxyl­ate

**DOI:** 10.1107/S1600536814023344

**Published:** 2014-11-05

**Authors:** Vladimir P. Zaytsev, Daria N. Orlova, Atash V. Gurbanov, Fedor I. Zubkov, Victor N. Khrustalev

**Affiliations:** a Organic Chemistry Department, Peoples’ Friendship University of Russia, Miklukho-Maklaya St 6, Moscow 117198, Russia; b Baku State University, Z. Khalilov St 23, Baku AZ-1148, Azerbaijan; c X-Ray Structural Centre, A.N. Nesmeyanov Institute of Organoelement Compounds, Russian Academy of Sciences, 28 Vavilov St, B-334, Moscow 119991, Russian Federation

**Keywords:** crystals structure, isoindolyl­pyrroles, benzodiazepines, IMDAF reaction, hydrogen bonds

## Abstract

The title compound, C_21_H_18_N_2_O_4_, obtained as a racemate, contains a novel heterocyclic system, *viz.* isoindolo[1,2-*c*]pyrrolo­[1,2-*a*][1,4]benzodiazepine. The central diazepane ring has a distorted boat conformation with two phenyl­ene-fused and one methine C atom deviating by 0.931 (1), 0.887 (1) and 0.561 (1) Å, respectively, from the mean plane of the rest of the ring. The γ-lactone ring has an envelope conformation, with the C atom opposite to amide bond deviating by 0.355 (1) Å from its plane. In the crystal, mol­ecules form centrosymmetric dimers through pairs of C—H⋯O hydrogen bonds.

## Related literature   

For the synthesis of pyrrolo­[1,2-*a*][1,4]benzodiazepine, see: Raines *et al.* (1976 ▶). For reviews on intra­molecular cyclo­addition reactions of α,β-unsaturated acid anhydrides to furfuryl­amines (IMDAF reactions), see: Vogel *et al.* (1999 ▶); Zubkov *et al.* (2005 ▶). For related compounds, see: Zubkov *et al.* (2009 ▶, 2014 ▶); Zubkov, Galeev *et al.* (2010 ▶); Zubkov, Zaitsev *et al.* (2010 ▶); Zaytsev *et al.* (2012 ▶, 2013 ▶); Toze *et al.* (2011 ▶).
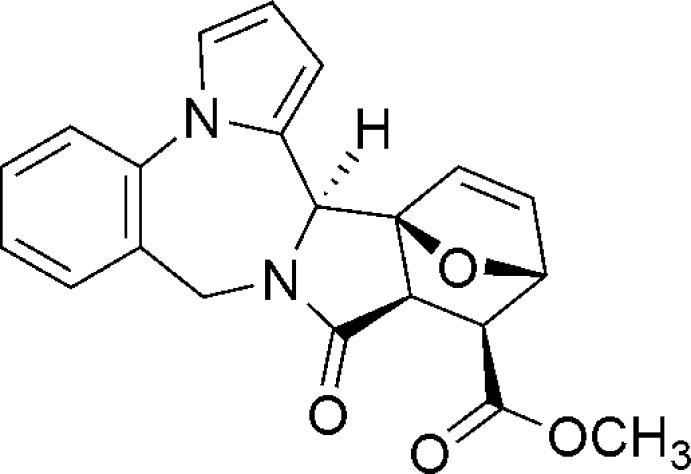


## Experimental   

### Crystal data  

C_21_H_18_N_2_O_4_
*M* *_r_* = 362.37
Monoclinic, 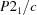

*a* = 8.1565 (3) Å
*b* = 14.2567 (5) Å
*c* = 14.6664 (5) Å
β = 98.210 (1)°
*V* = 1688.00 (10) Å^3^
*Z* = 4
Mo *K*α radiation
μ = 0.10 mm^−1^
*T* = 100 K
0.30 × 0.30 × 0.30 mm

### Data collection  

Bruker APEX DUO CCD diffractometer
Absorption correction: multi-scan (*SADABS*; Bruker, 2003 ▶) *T*_min_ = 0.971, *T*_max_ = 0.971
25733 measured reflections
6162 independent reflections
5097 reflections with *I* > 2σ(*I*)
*R*_int_ = 0.029

### Refinement  

*R*[*F* ^2^ > 2σ(*F* ^2^)] = 0.043
*wR*(*F* ^2^) = 0.119
*S* = 1.00
6162 reflections
245 parameters
H-atom parameters constrained
Δρ_max_ = 0.47 e Å^−3^
Δρ_min_ = −0.27 e Å^−3^

### 

Data collection: *APEX2* (Bruker, 2005 ▶); cell refinement: *SAINT* (Bruker, 2001 ▶); data reduction: *SAINT*; program(s) used to solve structure: *SHELXTL* (Sheldrick, 2008 ▶); program(s) used to refine structure: *SHELXTL*; molecular graphics: *SHELXTL*; software used to prepare material for publication: *SHELXTL*.

## Supplementary Material

Crystal structure: contains datablock(s) global, I. DOI: 10.1107/S1600536814023344/ld2132sup1.cif

Structure factors: contains datablock(s) I. DOI: 10.1107/S1600536814023344/ld2132Isup2.hkl

**Fig. 1.**
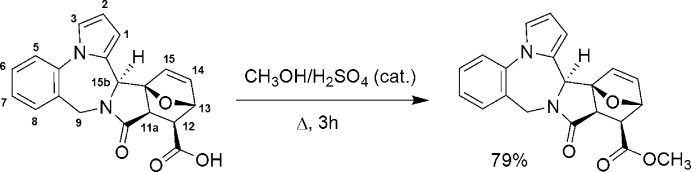
Synthesis of methyl 11-oxo-11,11a,12,13-tetrahydro-9H,15b-H-13,15a-epoxyisoindolo[1,2c]pyrrolo[1,2a][1,4]benzodiazepine-12-carboxylate.

H H c a . DOI: 10.1107/S1600536814023344/ld2132fig1.tif
Synthesis of methyl 11-oxo-11,11a,12,13-tetra­hydro-9*H*,15b-*H*-13,15a-ep­oxy­isoindolo[1,2*c*]pyrrolo­[1,2*a*][1,4]benzodiazepine-12-carboxyl­ate.

**Fig. 2.**
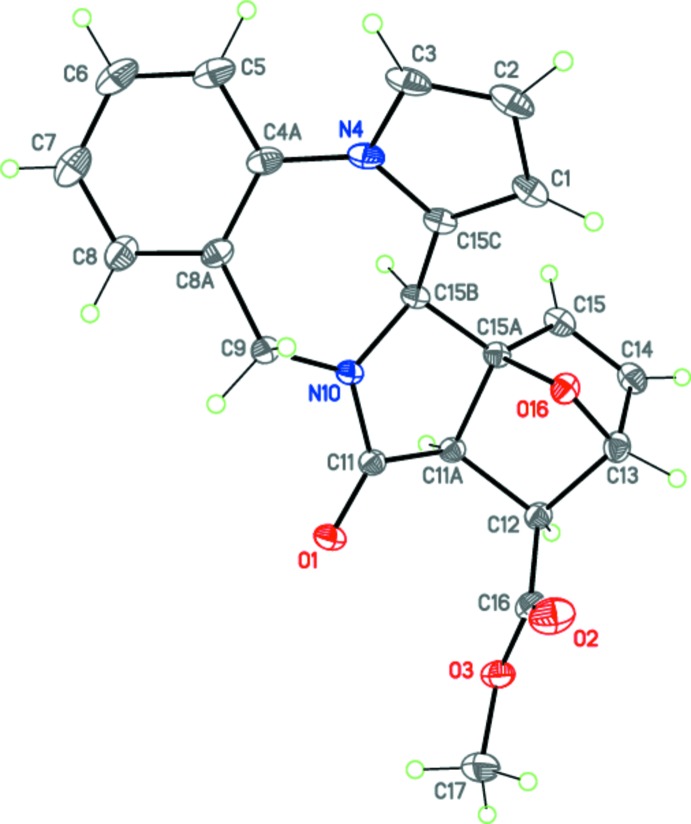
Molecular structure of (I). Displacement ellipsoids are shown at the 50% probability level. H atoms are depicted as small spheres of arbitrary radius.

. DOI: 10.1107/S1600536814023344/ld2132fig2.tif
Mol­ecular structure of (I). Displacement ellipsoids are shown at the 50% probability level. H atoms are depicted as small spheres of arbitrary radius.

**Fig. 3.**
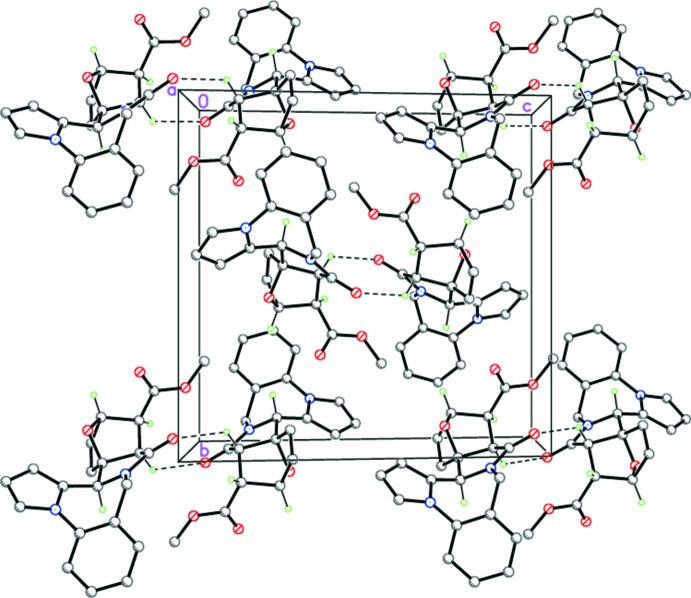
A portion of crystal packing of I along the crystallographic a axis demonstrating the centrosymmetric H-bonded dimers. Only hydrogen atoms at the asymmetric centers are shown. Dashed lines indicate the weak intermolecular C—H···O hydrogen bonds.

I a . DOI: 10.1107/S1600536814023344/ld2132fig3.tif
A portion of crystal packing of **I** along the crystallographic *a* axis demonstrating the centrosymmetric H-bonded dimers. Only hydrogen atoms at the asymmetric centers are shown. Dashed lines indicate the weak inter­molecular C—H⋯O hydrogen bonds.

CCDC reference: 903490

Additional supporting information:  crystallographic information; 3D view; checkCIF report

## Figures and Tables

**Table 1:** Hydrogen-bond geometry (, )

*D*H*A*	*D*H	H*A*	*D* *A*	*D*H*A*
C11*A*H11*A*O1^i^	1.00	2.54	3.2663(12)	129

Symmetry code: (i) 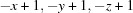.

## supplementary crystallographic information

### S1. Chemical context

In the last few years, our group has developed an effective strategy for the synthesis of 3,6a-ep­oxy­iso­indoles annulated withvarious heterocycles (Zubkov *et al.* 2009, 2014; Zubkov, Galeev *et al.* 2010; Zubkov, Zaitsev *et al.* 2010; Zaytsev *et al.* 2012, 2013). This strategy is based on the intra­molecular cyclo­addition reaction of α,β-unsaturated acid anhydrides to furfuryl­amines (IMDAF) (Vogel *et al.*, 1999; Zubkov *et al.*, 2005).

This article describes the synthesis (Raines *et al.*, 1976) and structure of a novel heterocyclic system – isoindolo[1,2-*c*]pyrrolo­[1,2-*a*][1,4]benzodiazepine, which can be easily obtained using the IMDAF reaction between maleic anhydride and 4-(2-furyl)-5,6-di­hydro-4*H*-pyrrolo­[1,2-*a*][1,4]benzodiazepine (Fig. 1). The resulting compound is an analogue of the previously described *rac*-methyl-11,13a-ep­oxy­pyrrolo­[2',1':3,4][1,4]diazepino[2,1-*a*]iso­indole-10-carboxyl­ate (Toze *et al.*, 2011).

### S2. Structural commentary

The title compound, C_21_H_18_N_2_O_4_ (I), includes a fused hexacyclic system containing four five-membered rings (pyrrole, 2-pyrrolidinone, tetra­hydro­furan and di­hydro­furan), one six-membered ring (benzene) and one seven-membered ring (1,4-diazepane) (Fig. 2). The pyrrole and benzene rings are planar; the 2-pyrrolidinone, tetra­hydro­furan and di­hydro­furan five-membered rings have the usual *envelope* conformations, and the central seven-membered diazepane ring adopts a *boat* conformation with the base plane composed from the N4, C15C, N10 and C9 atoms. The C4A, C8A and C15B atoms deviate from this base plane outward the bridge oxygen atom. As a consequence, the inter­plane angle between the *boat* bottom of the diazepane ring (N4/C9/N10/C15C) and the base plane of the central pyrrolidinone ring (N10/C11/C11A/C15B) is 19.80 (5)°. The nitro­gen N4 and N10 atoms have trigonal–planar geometry (sums of the bond angles are 360.0 (2) and 360.0 (3)°, respectively).

The molecule of I possesses five asymmetric centers at the C11A, C12, C13, C15A and C15B carbon atoms and can potentially have numerous diastereomers. The crystal of I is racemic and consists of enanti­omeric pairs with the following relative configuration of the centers: 11A*R**, 12*S**, 13*R**, 15A*S**, 15B*S**.

### S3. Supra­molecular features

In the crystal of I, the molecules form the centrosymmetric dimers by the two weak inter­molecular C11A—H11A···O1^i^ hydrogen bonds [C···O 3.2663 (12) Å, H···O 2.54, C—H···O 129°, Table 1, Fig. 3]. The crystal packing of the dimers is stacked along the *a* axis (Fig. 3). Symmetry code: (i) (1–*x*, 1–*y*, 1–*z*).

### S4. Synthesis and crystallization

A solution of the initial acid (0.5 g, 1.4 mmol) in methanol (40 mL) was refluxed for 3 h in the presence of catalytic amount of concentrated H_2_SO_4_ (monitoring by TLC until disappearance of the starting compound sport, eluent – EtOAc, Sorbfil). At the end of the reaction, the clear green solution was poured into water (100 mL) and extracted with CH_2_Cl_2_ (3×50 mL). The extract was dried over MgSO_4_ and concentrated in *vacuo*. The crude ester was recrystallized from a mixture of EtOH–DMF to give the title compound as yellow prisms. Yield is 0.4 g (79%). The single crystals of the product were obtained by slow crystallization from EtOH–DMF. *M.p*. = 492–493 K. IR (KBr), ν (cm^–1^): 1739, 1692; ^1^H NMR (CDCl_3_, 400 MHz, 300 K): δ = 2.76 (d, 1H, H11A, *J*_11A,12_ = 8.7), 2.91 (d, 1H, H12, *J*_11A,12_ = 8.7), 3.79 (s, 3H, CO_2_*Me*), 3.96 (d, 1H, H9B, *J*_9A,9B_ = 13.9), 4.95 (d, 1H, H9A, *J*_9A,9B_ = 13.9), 5.00 (s, 1H, H15B), 5.32 (d, 1H, H13, *J*_13,14_ = 1.8), 6.31 (dd, 1H, H2, *J*_2,3_ = 2.8, *J*_1,2_ = 3.7), 6.47 (d, 1H, H15, *J*_14,15_ = 5.9), 6.52 (dd, 1H, H14, *J*_13,14_ = 1.8, *J*_14,15_ = 5.9), 6.56 (dd, 1H, H1, *J*_1,3_ = 1.4, *J*_1,2_ = 3.7), 6.55 (dd, 1H, H3, *J*_1,3_ = 1.4, *J*_2,3_ = 2.8), 7.30–7.49 (m, 4H, C_6_*H*_4_). ^13^C NMR (CDCl_3_, 100 MHz, 300 K): δ = 43.5 (C9), 45.2, 51.5, 52.1, 53.7 (C11a, C12, C15B, CO_2_*Me*), 81.7 (C13), 90.2 (C15a), 109.9, 110.9 (C1, C2), 121.2, 123.2, 125.3, 127.0, 127.6, 129.8, 130.8, 134.3, 137.4, 140.1 (*C*_6_H_4_, C3, C14, C15, C15C), 168.4, 172.2 (N*C*O, *C*O_2_Me). EI–MS (70 eV) *m/z* (*rel*. intensity): 362 [*M*]^+^ (20), 249 (60),233 (32), 191 (19), 181 (54), 167 (31), 154 ( 73), 121 (21), 113 (100), 85 (38), 59 (29). Anal. Calcd for C_21_H_18_N_2_O_4_: C, 69.60; H, 5.01; N, 7.73. Found: C, 69.88; H, 4.89; N, 7.66.

### S5. Refinement

Crystal data, data collection and structure refinement details are summarized in Table 1. All hydrogen atoms were placed in the calculated positions with C—H = 0.95 (aryl-H), 0.98 (methyl-H), 0.99 (methyl­ene-H), and 1.00 (methine-H) Å and refined in the riding model with fixed isotropic displacement parameters: *U*_iso_(H) = 1.5*U*_eq_(C) for the CH_3_ group and *U*_iso_(H) = 1.2*U*_eq_(C) for the other groups. Positions of the hydrogen atoms of the methyl group were optimized rotationally.

### Crystal data

**Table:**

C_21_H_18_N_2_O_4_	*F*(000) = 760
*M**_r_* = 362.37	*D*_x_ = 1.426 Mg m^−^^3^
Monoclinic, *P*2_1_/*c*	Mo *K*α radiation, λ = 0.71073 Å
Hall symbol: -P 2ybc	Cell parameters from 8798 reflections
*a* = 8.1565 (3) Å	θ = 2.5–32.6°
*b* = 14.2567 (5) Å	µ = 0.10 mm^−^^1^
*c* = 14.6664 (5) Å	*T* = 100 K
β = 98.210 (1)°	Prism, yellow
*V* = 1688.00 (10) Å^3^	0.30 × 0.30 × 0.30 mm
*Z* = 4	

### Data collection

**Table:**

Bruker APEX DUO CCD diffractometer	6162 independent reflections
Radiation source: fine-focus sealed tube	5097 reflections with *I* > 2σ(*I*)
Graphite monochromator	*R*_int_ = 0.029
φ and ω scans	θ_max_ = 32.6°, θ_min_ = 2.0°
Absorption correction: multi-scan (*SADABS*; Bruker, 2003)	*h* = −12→12
*T*_min_ = 0.971, *T*_max_ = 0.971	*k* = −21→21
25733 measured reflections	*l* = −22→22

### Refinement

**Table:**

Refinement on *F*^2^	Primary atom site location: structure-invariant direct methods
Least-squares matrix: full	Secondary atom site location: difference Fourier map
*R*[*F*^2^ > 2σ(*F*^2^)] = 0.043	Hydrogen site location: inferred from neighbouring sites
*wR*(*F*^2^) = 0.119	H-atom parameters constrained
*S* = 1.00	*w* = 1/[σ^2^(*F*_o_^2^) + (0.0646*P*)^2^ + 0.565*P*] where *P* = (*F*_o_^2^ + 2*F*_c_^2^)/3
6162 reflections	(Δ/σ)_max_ < 0.001
245 parameters	Δρ_max_ = 0.47 e Å^−^^3^
0 restraints	Δρ_min_ = −0.27 e Å^−^^3^

### Special details

**Table:**

Geometry. All esds (except the esd in the dihedral angle between two l.s. planes) are estimated using the full covariance matrix. The cell esds are taken into account individually in the estimation of esds in distances, angles and torsion angles; correlations between esds in cell parameters are only used when they are defined by crystal symmetry. An approximate (isotropic) treatment of cell esds is used for estimating esds involving l.s. planes.
Refinement. Refinement of F^2^ against ALL reflections. The weighted R-factor wR and goodness of fit S are based on F^2^, conventional R-factors R are based on F, with F set to zero for negative F^2^. The threshold expression of F^2^ > 2sigma(F^2^) is used only for calculating R-factors(gt) etc. and is not relevant to the choice of reflections for refinement. R-factors based on F^2^ are statistically about twice as large as those based on F, and R- factors based on ALL data will be even larger.

### Fractional atomic coordinates and isotropic or equivalent isotropic displacement parameters (Å^2^)

**Table:**

	*x*	*y*	*z*	*U*_iso_*/*U*_eq_	
O1	0.27309 (9)	0.45215 (5)	0.53193 (5)	0.01848 (14)	
O2	0.47579 (12)	0.27298 (6)	0.63472 (6)	0.02842 (18)	
O3	0.60524 (10)	0.32277 (5)	0.51730 (5)	0.02255 (16)	
C1	0.31059 (13)	0.53225 (7)	0.90588 (6)	0.01946 (18)	
H1	0.4020	0.4912	0.9216	0.023*	
C2	0.19108 (14)	0.55791 (9)	0.96356 (7)	0.0242 (2)	
H2	0.1883	0.5366	1.0247	0.029*	
C3	0.08144 (14)	0.61843 (8)	0.91546 (7)	0.0228 (2)	
H3	−0.0107	0.6469	0.9374	0.027*	
N4	0.12777 (10)	0.63120 (6)	0.82896 (6)	0.01710 (16)	
C4A	0.04475 (12)	0.68950 (7)	0.75861 (7)	0.01735 (17)	
C5	−0.02093 (13)	0.77501 (7)	0.78280 (8)	0.0232 (2)	
H5	−0.0068	0.7944	0.8454	0.028*	
C6	−0.10691 (14)	0.83162 (8)	0.71507 (9)	0.0274 (2)	
H6	−0.1560	0.8884	0.7317	0.033*	
C7	−0.12110 (15)	0.80554 (8)	0.62343 (9)	0.0279 (2)	
H7	−0.1745	0.8460	0.5769	0.033*	
C8	−0.05706 (14)	0.71985 (8)	0.59930 (8)	0.0227 (2)	
H8	−0.0687	0.7021	0.5363	0.027*	
C8A	0.02398 (12)	0.65967 (7)	0.66631 (7)	0.01659 (17)	
C9	0.07294 (11)	0.56233 (7)	0.63935 (6)	0.01498 (16)	
H9A	0.0174	0.5155	0.6743	0.018*	
H9B	0.0334	0.5525	0.5730	0.018*	
N10	0.25065 (10)	0.54661 (6)	0.65654 (5)	0.01399 (14)	
C11	0.33580 (12)	0.49482 (6)	0.60081 (6)	0.01406 (16)	
C11A	0.51928 (11)	0.50475 (6)	0.63808 (6)	0.01349 (15)	
H11A	0.5705	0.5566	0.6059	0.016*	
C12	0.63419 (12)	0.41731 (6)	0.64878 (6)	0.01520 (16)	
H12	0.7414	0.4322	0.6264	0.018*	
C13	0.66222 (12)	0.40784 (7)	0.75634 (6)	0.01691 (17)	
H13	0.6992	0.3444	0.7801	0.020*	
C14	0.77432 (12)	0.48870 (8)	0.79392 (7)	0.01889 (18)	
H14	0.8884	0.4852	0.8186	0.023*	
C15	0.67943 (12)	0.56536 (7)	0.78501 (6)	0.01747 (17)	
H15	0.7105	0.6277	0.8026	0.021*	
C15A	0.51170 (11)	0.53094 (6)	0.74072 (6)	0.01330 (15)	
C15B	0.35216 (11)	0.58535 (6)	0.73907 (6)	0.01326 (15)	
H15B	0.3746	0.6530	0.7277	0.016*	
C15C	0.26875 (12)	0.57808 (7)	0.82316 (6)	0.01505 (16)	
O16	0.50407 (9)	0.43775 (5)	0.77891 (5)	0.01577 (13)	
C16	0.55961 (12)	0.32965 (7)	0.60170 (7)	0.01805 (18)	
C17	0.53752 (19)	0.24502 (8)	0.46127 (9)	0.0329 (3)	
H17A	0.6124	0.2289	0.4170	0.049*	
H17B	0.4290	0.2625	0.4280	0.049*	
H17C	0.5252	0.1908	0.5008	0.049*	

### Atomic displacement parameters (Å^2^)

**Table:**

	*U*^11^	*U*^22^	*U*^33^	*U*^12^	*U*^13^	*U*^23^
O1	0.0207 (3)	0.0205 (3)	0.0142 (3)	0.0013 (3)	0.0025 (2)	−0.0047 (2)
O2	0.0349 (5)	0.0188 (4)	0.0335 (4)	−0.0061 (3)	0.0117 (4)	−0.0013 (3)
O3	0.0306 (4)	0.0187 (3)	0.0186 (3)	0.0031 (3)	0.0043 (3)	−0.0035 (3)
C1	0.0229 (4)	0.0227 (4)	0.0130 (4)	−0.0056 (4)	0.0032 (3)	−0.0013 (3)
C2	0.0276 (5)	0.0331 (6)	0.0132 (4)	−0.0092 (4)	0.0074 (4)	−0.0036 (4)
C3	0.0234 (5)	0.0315 (5)	0.0158 (4)	−0.0079 (4)	0.0102 (4)	−0.0082 (4)
N4	0.0178 (4)	0.0196 (4)	0.0152 (3)	−0.0034 (3)	0.0069 (3)	−0.0047 (3)
C4A	0.0155 (4)	0.0161 (4)	0.0219 (4)	−0.0024 (3)	0.0076 (3)	−0.0039 (3)
C5	0.0196 (4)	0.0188 (4)	0.0329 (5)	−0.0022 (3)	0.0096 (4)	−0.0094 (4)
C6	0.0217 (5)	0.0158 (4)	0.0462 (7)	0.0012 (4)	0.0105 (5)	−0.0055 (4)
C7	0.0257 (5)	0.0190 (5)	0.0397 (6)	0.0049 (4)	0.0073 (5)	0.0042 (4)
C8	0.0232 (5)	0.0204 (4)	0.0254 (5)	0.0052 (4)	0.0066 (4)	0.0033 (4)
C8A	0.0159 (4)	0.0150 (4)	0.0200 (4)	0.0007 (3)	0.0065 (3)	−0.0004 (3)
C9	0.0147 (4)	0.0152 (4)	0.0152 (4)	0.0014 (3)	0.0023 (3)	−0.0014 (3)
N10	0.0146 (3)	0.0158 (3)	0.0117 (3)	0.0013 (3)	0.0023 (2)	−0.0021 (3)
C11	0.0170 (4)	0.0137 (4)	0.0121 (3)	0.0013 (3)	0.0043 (3)	0.0009 (3)
C11A	0.0152 (4)	0.0133 (4)	0.0127 (3)	0.0005 (3)	0.0043 (3)	0.0005 (3)
C12	0.0164 (4)	0.0146 (4)	0.0151 (4)	0.0012 (3)	0.0041 (3)	0.0008 (3)
C13	0.0168 (4)	0.0183 (4)	0.0161 (4)	0.0022 (3)	0.0036 (3)	0.0033 (3)
C14	0.0164 (4)	0.0258 (5)	0.0144 (4)	−0.0008 (3)	0.0022 (3)	0.0004 (3)
C15	0.0162 (4)	0.0215 (4)	0.0150 (4)	−0.0044 (3)	0.0034 (3)	−0.0015 (3)
C15A	0.0151 (4)	0.0130 (4)	0.0123 (3)	−0.0013 (3)	0.0036 (3)	0.0006 (3)
C15B	0.0154 (4)	0.0133 (3)	0.0115 (3)	−0.0017 (3)	0.0035 (3)	−0.0015 (3)
C15C	0.0168 (4)	0.0158 (4)	0.0132 (4)	−0.0040 (3)	0.0046 (3)	−0.0029 (3)
O16	0.0173 (3)	0.0152 (3)	0.0157 (3)	0.0002 (2)	0.0054 (2)	0.0037 (2)
C16	0.0198 (4)	0.0150 (4)	0.0195 (4)	0.0041 (3)	0.0036 (3)	−0.0004 (3)
C17	0.0497 (8)	0.0209 (5)	0.0256 (5)	0.0033 (5)	−0.0037 (5)	−0.0070 (4)

### Geometric parameters (Å, º)

**Table:**

O1—C11	1.2267 (11)	C9—H9A	0.9900
O2—C16	1.2039 (13)	C9—H9B	0.9900
O3—C16	1.3460 (12)	N10—C11	1.3629 (11)
O3—C17	1.4420 (14)	N10—C15B	1.4729 (11)
C1—C15C	1.3773 (13)	C11—C11A	1.5247 (13)
C1—C2	1.4266 (15)	C11A—C12	1.5541 (13)
C1—H1	0.9500	C11A—C15A	1.5607 (13)
C2—C3	1.3644 (17)	C11A—H11A	1.0000
C2—H2	0.9500	C12—C16	1.5129 (14)
C3—N4	1.3866 (12)	C12—C13	1.5672 (13)
C3—H3	0.9500	C12—H12	1.0000
N4—C15C	1.3896 (13)	C13—O16	1.4413 (12)
N4—C4A	1.4192 (13)	C13—C14	1.5247 (14)
C4A—C5	1.3976 (14)	C13—H13	1.0000
C4A—C8A	1.4059 (13)	C14—C15	1.3349 (15)
C5—C6	1.3897 (17)	C14—H14	0.9500
C5—H5	0.9500	C15—C15A	1.5102 (13)
C6—C7	1.3834 (18)	C15—H15	0.9500
C6—H6	0.9500	C15A—O16	1.4466 (11)
C7—C8	1.3941 (15)	C15A—C15B	1.5122 (13)
C7—H7	0.9500	C15B—C15C	1.4948 (12)
C8—C8A	1.3976 (14)	C15B—H15B	1.0000
C8—H8	0.9500	C17—H17A	0.9800
C8A—C9	1.5123 (13)	C17—H17B	0.9800
C9—N10	1.4528 (12)	C17—H17C	0.9800
			
C16—O3—C17	116.50 (9)	C11—C11A—H11A	110.8
C15C—C1—C2	107.24 (10)	C12—C11A—H11A	110.8
C15C—C1—H1	126.4	C15A—C11A—H11A	110.8
C2—C1—H1	126.4	C16—C12—C11A	114.78 (8)
C3—C2—C1	107.97 (9)	C16—C12—C13	112.31 (8)
C3—C2—H2	126.0	C11A—C12—C13	99.79 (7)
C1—C2—H2	126.0	C16—C12—H12	109.9
C2—C3—N4	108.18 (9)	C11A—C12—H12	109.9
C2—C3—H3	125.9	C13—C12—H12	109.9
N4—C3—H3	125.9	O16—C13—C14	101.74 (8)
C3—N4—C15C	108.69 (9)	O16—C13—C12	101.47 (7)
C3—N4—C4A	125.31 (9)	C14—C13—C12	107.01 (8)
C15C—N4—C4A	126.00 (8)	O16—C13—H13	115.0
C5—C4A—C8A	120.69 (10)	C14—C13—H13	115.0
C5—C4A—N4	119.12 (9)	C12—C13—H13	115.0
C8A—C4A—N4	120.15 (8)	C15—C14—C13	105.92 (8)
C6—C5—C4A	119.85 (10)	C15—C14—H14	127.0
C6—C5—H5	120.1	C13—C14—H14	127.0
C4A—C5—H5	120.1	C14—C15—C15A	104.65 (8)
C7—C6—C5	120.14 (10)	C14—C15—H15	127.7
C7—C6—H6	119.9	C15A—C15—H15	127.7
C5—C6—H6	119.9	O16—C15A—C15	102.61 (7)
C6—C7—C8	119.99 (11)	O16—C15A—C15B	113.13 (7)
C6—C7—H7	120.0	C15—C15A—C15B	124.39 (8)
C8—C7—H7	120.0	O16—C15A—C11A	99.42 (7)
C7—C8—C8A	121.07 (10)	C15—C15A—C11A	109.54 (7)
C7—C8—H8	119.5	C15B—C15A—C11A	105.06 (7)
C8A—C8—H8	119.5	N10—C15B—C15C	112.54 (7)
C8—C8A—C4A	118.12 (9)	N10—C15B—C15A	101.67 (7)
C8—C8A—C9	119.70 (9)	C15C—C15B—C15A	116.31 (8)
C4A—C8A—C9	121.96 (9)	N10—C15B—H15B	108.7
N10—C9—C8A	112.98 (8)	C15C—C15B—H15B	108.7
N10—C9—H9A	109.0	C15A—C15B—H15B	108.7
C8A—C9—H9A	109.0	C1—C15C—N4	107.93 (8)
N10—C9—H9B	109.0	C1—C15C—C15B	132.45 (9)
C8A—C9—H9B	109.0	N4—C15C—C15B	119.39 (8)
H9A—C9—H9B	107.8	C13—O16—C15A	95.36 (7)
C11—N10—C9	124.11 (8)	O2—C16—O3	124.65 (10)
C11—N10—C15B	114.91 (8)	O2—C16—C12	125.82 (9)
C9—N10—C15B	120.97 (7)	O3—C16—C12	109.51 (8)
O1—C11—N10	125.05 (9)	O3—C17—H17A	109.5
O1—C11—C11A	127.86 (8)	O3—C17—H17B	109.5
N10—C11—C11A	107.03 (7)	H17A—C17—H17B	109.5
C11—C11A—C12	120.57 (8)	O3—C17—H17C	109.5
C11—C11A—C15A	101.30 (7)	H17A—C17—H17C	109.5
C12—C11A—C15A	101.58 (7)	H17B—C17—H17C	109.5
			
C15C—C1—C2—C3	−0.39 (12)	C13—C14—C15—C15A	−0.95 (10)
C1—C2—C3—N4	0.35 (12)	C14—C15—C15A—O16	33.34 (9)
C2—C3—N4—C15C	−0.19 (12)	C14—C15—C15A—C15B	163.20 (8)
C2—C3—N4—C4A	−179.84 (9)	C14—C15—C15A—C11A	−71.57 (9)
C3—N4—C4A—C5	37.78 (14)	C11—C11A—C15A—O16	85.68 (7)
C15C—N4—C4A—C5	−141.80 (10)	C12—C11A—C15A—O16	−39.10 (8)
C3—N4—C4A—C8A	−139.92 (10)	C11—C11A—C15A—C15	−167.24 (7)
C15C—N4—C4A—C8A	40.50 (14)	C12—C11A—C15A—C15	67.98 (9)
C8A—C4A—C5—C6	−0.39 (15)	C11—C11A—C15A—C15B	−31.51 (8)
N4—C4A—C5—C6	−178.07 (9)	C12—C11A—C15A—C15B	−156.29 (7)
C4A—C5—C6—C7	−2.93 (17)	C11—N10—C15B—C15C	−138.43 (8)
C5—C6—C7—C8	3.57 (18)	C9—N10—C15B—C15C	40.32 (11)
C6—C7—C8—C8A	−0.88 (17)	C11—N10—C15B—C15A	−13.28 (10)
C7—C8—C8A—C4A	−2.36 (16)	C9—N10—C15B—C15A	165.47 (8)
C7—C8—C8A—C9	172.37 (10)	O16—C15A—C15B—N10	−80.04 (8)
C5—C4A—C8A—C8	2.98 (14)	C15—C15A—C15B—N10	154.51 (8)
N4—C4A—C8A—C8	−179.36 (9)	C11A—C15A—C15B—N10	27.37 (8)
C5—C4A—C8A—C9	−171.61 (9)	O16—C15A—C15B—C15C	42.57 (10)
N4—C4A—C8A—C9	6.05 (14)	C15—C15A—C15B—C15C	−82.88 (11)
C8—C8A—C9—N10	118.64 (10)	C11A—C15A—C15B—C15C	149.98 (8)
C4A—C8A—C9—N10	−66.84 (11)	C2—C1—C15C—N4	0.27 (11)
C8A—C9—N10—C11	−143.31 (9)	C2—C1—C15C—C15B	174.54 (10)
C8A—C9—N10—C15B	38.06 (11)	C3—N4—C15C—C1	−0.05 (11)
C9—N10—C11—O1	−3.19 (15)	C4A—N4—C15C—C1	179.59 (9)
C15B—N10—C11—O1	175.52 (9)	C3—N4—C15C—C15B	−175.20 (8)
C9—N10—C11—C11A	174.16 (8)	C4A—N4—C15C—C15B	4.44 (14)
C15B—N10—C11—C11A	−7.13 (10)	N10—C15B—C15C—C1	118.64 (11)
O1—C11—C11A—C12	−48.29 (13)	C15A—C15B—C15C—C1	1.93 (15)
N10—C11—C11A—C12	134.46 (8)	N10—C15B—C15C—N4	−67.62 (11)
O1—C11—C11A—C15A	−159.13 (9)	C15A—C15B—C15C—N4	175.67 (8)
N10—C11—C11A—C15A	23.61 (9)	C14—C13—O16—C15A	49.73 (8)
C11—C11A—C12—C16	12.35 (12)	C12—C13—O16—C15A	−60.58 (8)
C15A—C11A—C12—C16	123.04 (8)	C15—C15A—O16—C13	−51.04 (8)
C11—C11A—C12—C13	−107.91 (9)	C15B—C15A—O16—C13	172.50 (7)
C15A—C11A—C12—C13	2.78 (8)	C11A—C15A—O16—C13	61.58 (7)
C16—C12—C13—O16	−87.41 (9)	C17—O3—C16—O2	4.70 (15)
C11A—C12—C13—O16	34.64 (8)	C17—O3—C16—C12	−177.05 (9)
C16—C12—C13—C14	166.38 (8)	C11A—C12—C16—O2	−87.26 (12)
C11A—C12—C13—C14	−71.58 (9)	C13—C12—C16—O2	25.82 (14)
O16—C13—C14—C15	−31.71 (9)	C11A—C12—C16—O3	94.52 (9)
C12—C13—C14—C15	74.32 (9)	C13—C12—C16—O3	−152.41 (8)

### Hydrogen-bond geometry (Å, º)

**Table:**

*D*—H···*A*	*D*—H	H···*A*	*D*···*A*	*D*—H···*A*
C11*A*—H11*A*···O1^i^	1.00	2.54	3.2663 (12)	129

Symmetry code: (i) −*x*+1, −*y*+1, −*z*+1.
