# Supplementary figures and images for: Crystal strucutre of rac-methyl (11aR*,12S*,13R*,15aS*,15bS*)-11-oxo-11,11a,12,13-tetra­hydro-9H,15bH-13,15a-ep­oxy­isoindolo[1,2-c]pyrrolo[1,2-a][1,4]benzodiazepine-12-carboxyl­ate
Source: Acta Crystallogr Sect E Struct Rep Online. 2014 Nov 5;70(Pt 12):o1225–6. doi: 10.1107/S1600536814023344 (PMC4257400; doi:10.1107/S1600536814023344)

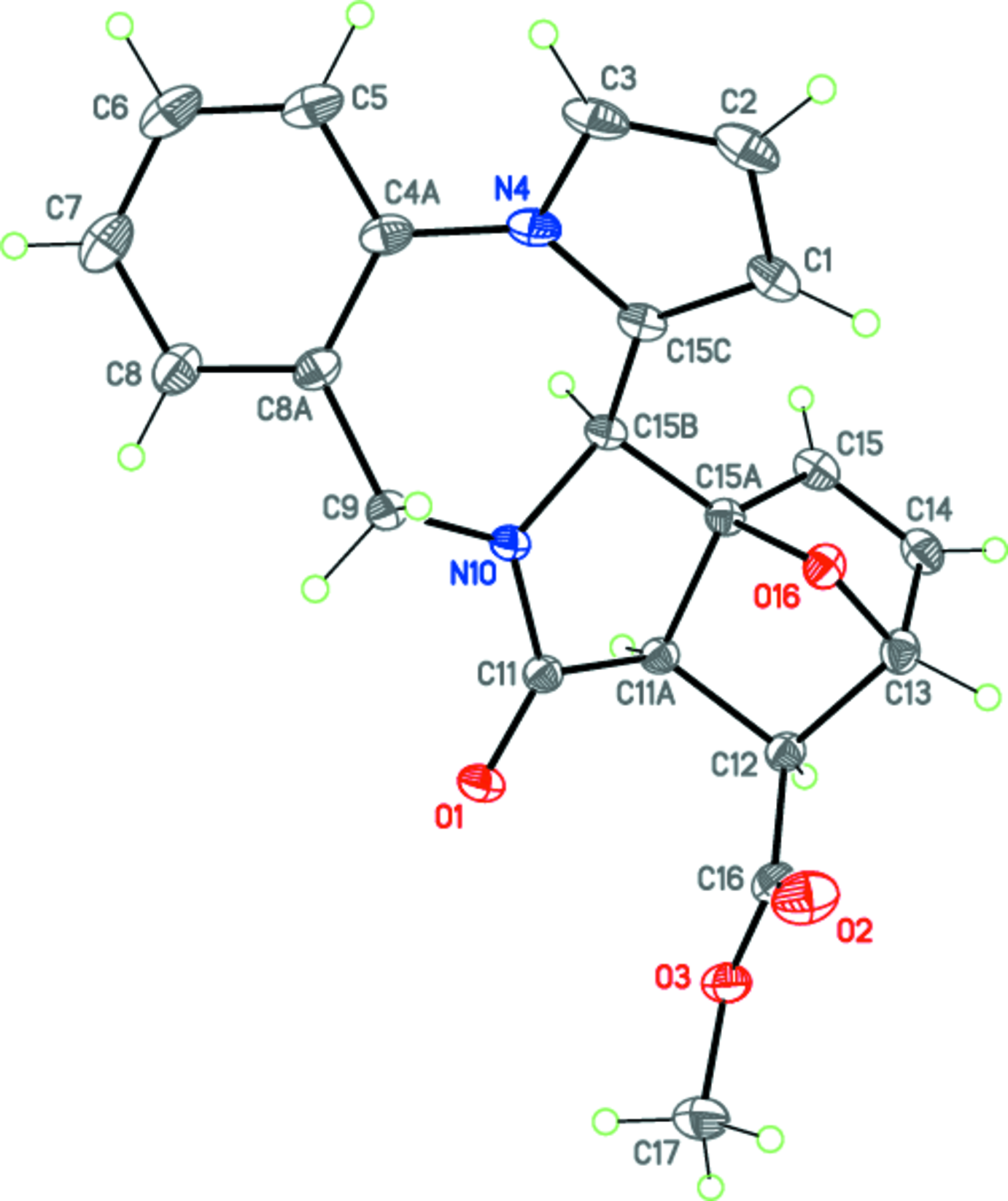

Supplement: Supplementary file 4 [file e-70-o1225-fig2.tif]

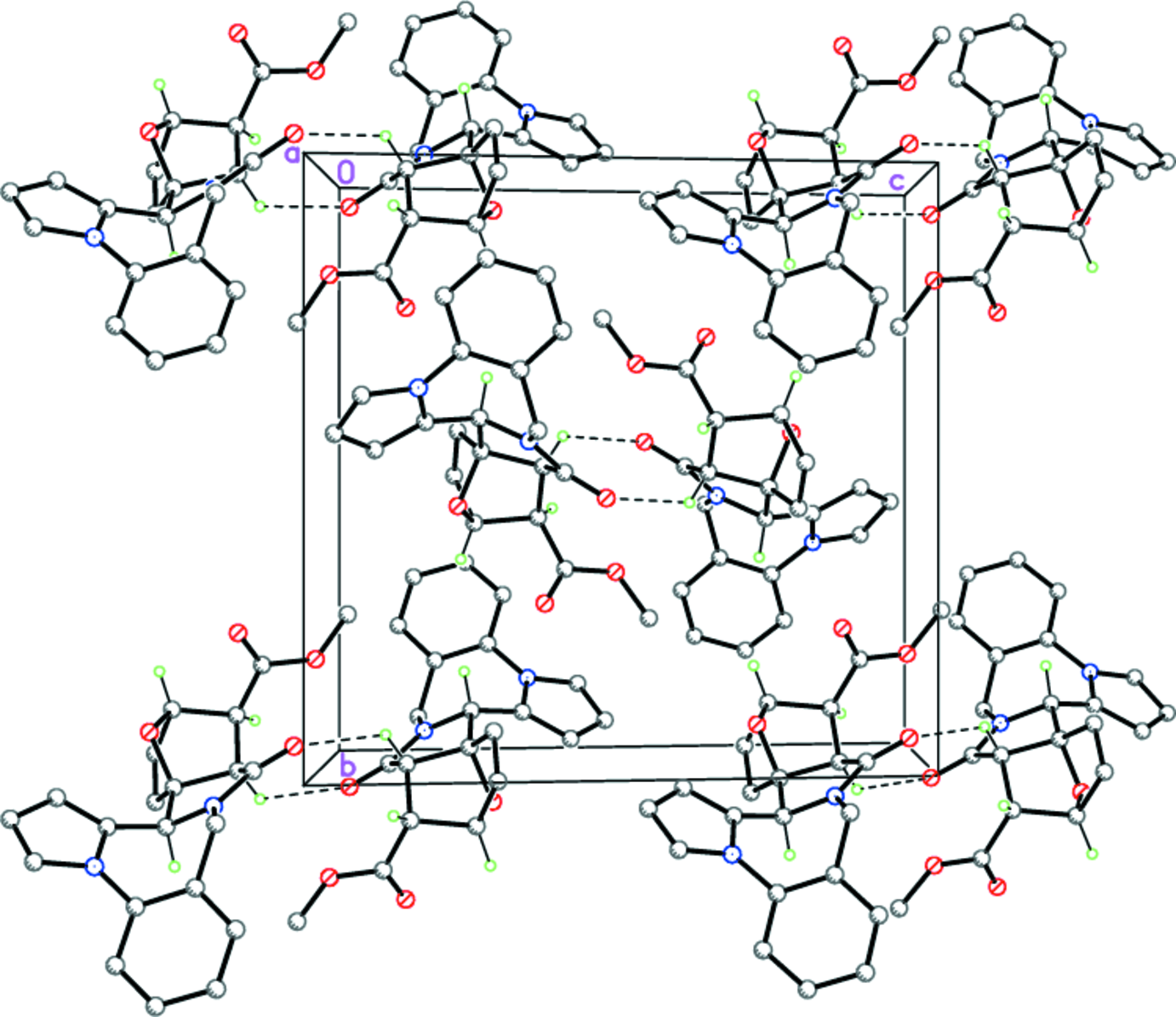

Supplement: Supplementary file 5 [file e-70-o1225-fig3.tif]
